# Hahnemann’s “Chronic Diseases”

**Published:** 1885-02

**Authors:** C. L. Swift


### HAHNEMANN'S "CHRONIC DISEASES."

C. L. SWIFT, M. D.

[Read before the Central New York Homœopathic Medical Society.]

To-day our subject for discussion is Hahnemann's *Chronic Diseases*—whether his theory or views with regard to the causes of chronic diseases be true or not.

In order to discuss any subject intelligently, we must know all we can about it, so that we may arrive at the proper understanding of the subject.

Diseases are divided into two classes: acute and chronic. Chronic diseases are those which continue on and on, even to the death of the patient.

The causes of chronic diseases are undoubtedly various.

Different persons have different views with regard to this *something* within the organism which seems to pervade and deteriorate the life-giving principle, and which, without the aid of remedies, continues to increase to the end of life.

Hahnemann's views of the nature of chronic diseases were these. "All chronic diseases originate from three distinct miasms, viz.: syphilis, sycosis, and psora, and that seven-eighths of all chronic diseases arise from psora."

Hahnemann found in the treatment of this class of chronic diseases that many of his patients were not cured by the remedy selected according to the symptoms of the patient, and that some he even failed to relieve.

In comparing the treatment of acute and chronic diseases, the wonderful results in the former class of diseases with the failures in the latter, he asks himself these questions: "What, then, was the reason why the continued homœopathic treatment of the non-venereal chronic diseases should have been so unsuccessful? Why should Homœopathy have failed in thousands of cases to cure such chronic ailments thoroughly and forever?"

He also says, "Ever since the years 1816 and 1817 I have been employed day and night to discover the reason why the homœopathic remedies which were then known did not effect a true cure of the above-named chronic diseases. I tried to obtain a more correct, and, if possible, a completely correct idea of the true nature of those thousands of chronic ailments which remained uncured, in spite of the incontrovertible truth of the homœopathic doctrine, when, behold! the Giver of all good permitted me about that time to solve the sublime problem for the benefit of mankind after unceasing meditation, indefatigable research, careful observation, and the most accurate experiments. * * * * I had reached this point when my investigations and observations upon non-venereal chronic patients led me at once to perceive that a previously existing itch, which they often confessed to have had, was the cause why many diseases that appeared to be separate and coherent maladies should not be cured by homœopathic treatment.

"All the subsequent sufferings were dated from the time when the psoric eruption had manifested itself. In many of these chronic patients who were unwilling to confess having had the itch, or had been too careless to heed it, or had no recollections of it, I often discovered by careful inquiries that vestiges of the itch had shown themselves upon their bodies from time to time in the shape of small pustules or herpes, etc., as so many infallible symptoms of the chronic contagion.

"These circumstances, coupled with the fact that psoric eruptions which had been removed by evil practices, or by some other cause, were evidently followed in otherwise healthy persons by chronic ailments, having the like or similar symptoms, as had been observed both by other physicians and myself in an infinite number of cases, left me no doubt about the internal enemy which I had to combat in my medical treatment.

"This internal enemy I shall designate by the general term *psora*. It is an internal disease, a sort of internal itch, and may exist either with or without an eruption upon the skin. Little by little I discovered more adequate remedies against this internal disease from which sprang so many sufferings. From the relief which I obtained by their employment in cases where the patient had no recollection of the itch, I inferred that they resulted from a psora, which had been communicated to the patient in the cradle, or in some other way of which he had no recollection. By carefully inquiring of the parents or relatives I discovered that my suspicions were well founded."

Hartmann says in the introduction to his *Chronic Diseases*, in 1817 and 1818: "I had frequent opportunities of hearing patients examined by Hahnemann, who generally asked them whether they had had the itch. Since then I have never omitted asking a similar question, and frequently cured a chronic malady with Sulphur or Hepar. s., even before Hahnemann's *Chronic Diseases* had made its appearance."

Quoting again from Hahnemann's *Chronic Diseases*: "Though this *psora* is the *oldest, most universal*, and *most pernicious*

chronic miasmatic disease, yet it has been misapprehended more than any other.

"For thousands of years it has disfigured and tortured mankind; and during the last centuries it has become the cause of those thousands of incredibly different acute as well as chronic non-venereal diseases with which the civilized portion of mankind becomes more and more infected upon the whole inhabited globe.

"Psora is the oldest miasmatic chronic disease known. The oldest history of the oldest nations does not reach its origin.

"According to the most ancient historical writings which we possess, *psora* existed already, almost fully developed, in the earliest ages of mankind. Several varieties of *psora* have been already delineated by Moses three thousand four hundred years ago. At that time, however, and always afterward, among the Israelites *psora* appears to have especially infected the external parts of the body. This was also the case among the Greek barbarians, then among the Arabs, and finally in the uncivilized Europe of the Middle Ages.

"In the Middle Ages Europe was revisited for several centuries by the frightful *psora* of the Occidental countries, in the shape of a malignant erysipelas, called St. Anthony's fire. In the thirteenth century it assumed again the form of leprosy. * * * Toward the end of the fifteenth century it assumed the ordinary eruption of the itch."

Having given a brief résumé of Hahnemann's views on the nature and causes of chronic non-venereal diseases, we are better able to discuss them.

There are those in our ranks who reject this theory of Hahnemann and deny its truth in toto. Others think it is overdrawn, while others believe it as thoroughly as did Hahnemann.

Dr. Lilienthal, in the transactions of the Homœopathic Medical Society of the State of New York, 1870, page 450, says: "Looking at the etymology of the word psora, or even of the word scabies, we find in the works of Erasmus Willson, certainly an acknowledged authority in skin diseases, that the words eczema and psora mean one and the same thing—itch, itchiness—because of the necessity which is induced by its itchiness to rub or to scratch, the word (psocire) meaning to rub. This is the language of Hippocrates, and we all know how deeply the works of the sage of Kos were studied by Hahnemann."

Celsus designates the same diseased state by the Latin name scabies, from scabere, to scratch, or impetigo, from ab impetu agens, a breaking out of impetus, an involuntary scratching, and all the four terms, eczema, psora, scabies, impetigo, have originally been applied to one and the same disease. * * * Studying again the word psoriasis, from psora, we find it by different authors restricted to different chronic skin diseases, to the lepra alphos of the Greeks, to the lepra vulgaris, etc., and it would be unjust to confine the meaning of the word psora according to the teachings of Hahnemann to such narrow limits. That great and good man understood by the word psora and psoric dyscrasia that undefinable contamination of the blood so often found in our days that a healthy offspring is a *rara avis* in our civilized age.

Dunglison divides psora into "psora, the itch; psora agria, psoriasis inveterata; psora ebriorum, drunkard's itch; and psora leprosa, psoriasis, of which Dr. Willan has given names to eleven varieties."

That the name *itch*, which we apply to the disease scabies, was used by Hahnemann and others in a much broader sense cannot be doubted. That it not only included with itch eczema, but also the majority of skin diseases described by Dr. Willan.

In the *American Journal of Homœopathy*, June, 1854, Professor Henderson, in a reply to Dr. Simpson, shows that the psoric doctrine of Hahnemann means more than the scabies of to-day, and also that it antedates Hahnemann's time. He says: "Psora was an ancient term, used almost indiscriminately for every diversity of chronic and almost every kind of acute cutaneous disease."

Frederick Hoffman (an allopath), who laid "the basis of the pathology at present taught in the schools of medicine," after adverting to the occurrence of pains in the joints on the cessation of ulcers in the legs, also adds: "We have known likewise atrocious pains of the joints suddenly removed on the occurrence of psora or itch (psora vel scabie) having the character of white lepra." He adds: "Experience itself teaches this truth; for innumerable observations of the most credible authors exist which record that spasmodic asthma, inflammation of the joints, gout, and many other diseases have been removed on the appearance of the itch (scabies), and, on the other hand, have arisen on the itch being suppressed."

Autenreith (an allopath) says: "The most formidable, and in our country the most frequent, source of the chronic diseases of the adult are the itch eruptions badly treated by sulphur ointments, or by other active greasy applications."

Is there one who doubts the truth of this assertion? There are no skin diseases which can be treated lightly. Call them itch, psora, eczema, or what not, their suppression will always result disastrously to the patient.

In the year 1834, six years after Hahnemann had published his *Chronic Diseases*, M. Renucci, a young Corsican in Paris, proved to the world that the itch was caused by a parasite called acarus scabiei seu sarcoptes hominis. The old school considered this a great triumph, and that Hahnemann's psora theory was exploded.

Dr. Hering says in his preface to Hahnemann's *Chronic Diseases:* "The shallow opponents of Homœopathy, and we never had any other, pounced upon the theory of the psoric miasm with a view of attacking it with hollow and unmeaning sarcasms. Making psora identical with itch, they sneeringly pretended that according to Hahnemann's doctrine the itch was the primitive evil, and that this doctrine was akin to the doctrine of the original sin recognized by the Christian faith."

Dr. Hempel says in a foot-note: "As it would be absurd for a philosophical Christian to reject the doctrine of original sin, so it is absurd for any one who professes to have a clear perception of Homœopathy to reject the doctrine of an hereditary morbific miasm. * * * It is this principle which Hahnemann calls psora."

Dr. Wilkinson (member of the Royal College of Surgeons in England) says: "It is, however, in the eradication of chronic diseases and hereditary taints that Homœopathy promises, perhaps, the greatest of its benefits. On this subject the views of Hahnemann deserve the attention of philanthropists of every degree, while at the same time, they are highly interesting to the medical philosopher. Nay, there is a touch of the sublime about them such as only comes into the scientific spirit in its happiest moods.

"As Hahnemann teaches us of the true contagions that have come down with men from early days, we seem to hear the echoes of every myth that has struck us with significance before, from the Parsee dualism of Ahriman and Ormuzd to the blue-white Hela of Scandinavian faith.

"Nay, also, we are let into the under strata of that evil which throws out sulphurs and geysers in the human and inhuman worlds, and we cease to wonder that no cure comes when the pit of disease is so deep. What a chasteness of genius, too, in Hahnemann that, instead of swerving to speculations, he forced these corruptions through the outlets of his method of cure and thought nothing sacred enough for his attention but the recovery of the body from its ancient pests."

Raue says in his *Pathology:* "If we now take a glance over Hahnemann's masterly picture of what he calls psora, we shall at once perceive that under psora he did not understand acarus-itch solely, but gave a *tout ensemble* of chronic cutaneous affections in general. The child had to have a name, and psora was as good as eczema, impetigo, prurigo, or any other. It is just as true to-day that a suppression of cutaneous eruptions of various kinds will be followed by disastrous consequences upon the general system as it was true when Hahnemann and others observed it. Instead, then, of desiring to have Hahnemann's psora theory wiped out of the pages of Homœopathy as a disgraceful spot, we ought to be proud of our old master's keen observation."

Dr. Hering asks: "Why has a great number of homœopathic physicians neither recognized Hahnemann's theory of *psora* nor the specific character of the anti-psoric remedies? Why have some gone so far as to set the theory sneeringly aside and to decry the anti-psorics as less trustworthy than the other remedies?"

For the same reason that the astronomical discoveries of our Herschel are doubted by people who have no faith in the discoverer and are not able to verify his discoveries. To do this knowledge, instruments, talent, care, perseverance, opportunities and many other things are required. Not one of all these requisites can be found with those who are mere dabblers in practice, scribbling authors, opposing their own opinions and imaginations to facts and observations.

Or for the same reason that Ehrenberg's discoveries cannot be appreciated by those who have either no microscope or who have one which is not good, or who have one without understanding the difficult art of using it, or else who know how to use it but do not use it with the same exactness and carefulness as Ehrenberg, who discovered in the chalk-dust of visiting cards the shells of a new species of animals by simply making the cards transparent by means of the oil of turpentine.

Or lastly, for the simple reason that physicians find it more easy to write something for print than to observe nature, that it is more easy to impose upon people than to cure the sick, and because the greater number of physicians are affected with the delusion that things which they do not see do not exist.

Dr. James Chapman says in the *Homœopathic Times,* 1850: "We have, as medical men, to do with a sin-poisoned race; the sins of the fathers are reproduced in their offspring, diseases are transmitted, and a loathsome taint pervades the fountain of life. This taint Hahnemann calls the psoric.

"Psora is a general term for skin diseases; it is applied generally to what is commonly called the itch. But in Hahnemann's view of the subject it is traced back to the leprosy of the Hebrews, recorded in the Old Testament, to that of the Arabians, and to that once prevalent form of disease for which lazar-houses were erected in almost every town and city of Christendom.

"The lazar-houses have disappeared; the worst forms of leprosy are no longer seen in Christendom. Have we therefore escaped the pollution? We trow we have not escaped it. In another form it vitiates the blood of all the descendants of Japhet, and no doubt influences the health of the families of Shem and Ham.

"But we speak of Japhet—of Europeans and their offshoots. This is the psoric taint of which Hahnemann speaks.

"It is no small merit of that most wonderful man that he traced with patient industry this unseen, unnoticed taint to its old forms; that he marked it in the chronic diseases of the moderns; that he saw its plague-spot and tracked it in the snow of the ancients.

"Little do the Sybarites dream of the misery of the psoric-tainted when exposed to temptation.

"General Bonaparte had a skin-disease, contracted in his first Italian campaign; this was repelled by unguents. The feverish excitement and the occasional madness of the Emperor were due to this repelled skin-disease. After its repulsion he had epilepsy, and he died, when not old, of cancer of the stomach. Instances might be multiplied without end.

"We might speak of scrofula, the disease of England; of consumption and rickets, and its other various forms; of gout and rheumatism; of mania, epilepsy, catalepsy, and a list of nervous diseases. The psoric taint is the root of all."

Ever since the fall of our first parents in the Garden of Eden and the death of Abel by the hand of his brother Cain, through jealousy, sin and death have been the history of our race.

Disease had been transmitted from father to son, mother to daughter, families to families, friends and foes alike.

At times disease would rage with such fierceness that there were scarcely enough well persons to bury the dead. The stillness of the night was broken by the dead-cart and its echo, "Bring out your dead."

Medical men looked on utterly helpless to relieve. Years passed by, leaving increased science more perfected, but how was it with the art of healing? What a condition of things when one of the leading men of its ranks must say, " We must mend or end "!

During this time, when medical men seemed to fail, when disease and death were claiming their victims on every hand, " there was a cloud the size of a man's hand " appeared above the horizon. Slowly it grew. It was driven by the wind of oppression to other places, but still it grew. The sick that rested under its shadow were revived. New health and vigor returned. The balmy breezes of healing were wafted across the oceans, over the mountains, and through the valleys, and we to-day are enjoying the fruitage of Homœopathy. Its founder is gone, but, not like many of us, he still lives in his works. The truths that he gave to the world will live whether they be accepted or not.

In that wonderful work, the *Organon*, he carefully leads us from the visionary fields of the ancients into the clear light of the true art of healing.

In his *Chronic Diseases* he clears away the labyrinths of pathological hypothesis, shows us the nature of those diseases, whose name is *legion*, whose cause is due to a morbific taint he calls psora, and then, lastly, how to apply the true art of healing to cure and relieve this class of suffering.

This *psora*, this *mote* which so many have been trying to pluck out as a stain on the pages of Homœopathy, as they try to pick out the itch-bug, and claim that they hold it all on the point of their needle, if they would understand its wonderful revelation they must first pluck the beam of hypocrisy, superstition, and prejudice from their own minds, and then they will see clearly the wonderful comprehension of our Hahnemann.

